# Bioinformatics and Functional Assessment of Toxin-Antitoxin Systems in *Staphylococcus aureus*

**DOI:** 10.3390/toxins10110473

**Published:** 2018-11-14

**Authors:** Gul Habib, Qing Zhu, Baolin Sun

**Affiliations:** Division of Molecular Medicine, Hefei National Laboratory for Physical Sciences at Microscale, the CAS Key Laboratory of Innate Immunity and Chronic Disease, School of Life Sciences, University of Science and Technology of China, Hefei 230027, China; gulhabib@mail.ustc.edu.cn (G.H.); zhuqq@mail.ustc.edu.cn (Q.Z.)

**Keywords:** *Staphylococcus aureus*, toxin-antitoxin systems, HigBA, pathogenicity islands

## Abstract

*Staphylococcus aureus* is a nosocomial pathogen that can cause chronic to persistent infections. Among different mediators of pathogenesis, toxin-antitoxin (TA) systems are emerging as the most prominent. These systems are frequently studied in *Escherichia coli* and *Mycobacterial* species but rarely explored in *S. aureus*. In the present study, we thoroughly analyzed the *S. aureus* genome and screened all possible TA systems using the Rasta bacteria and toxin-antitoxin database. We further searched *E. coli* and *Mycobacterial* TA homologs and selected 67 TA loci as putative TA systems in *S. aureus*. The host inhibition of growth (HigBA) TA family was predominantly detected in *S. aureus*. In addition, we detected seven pathogenicity islands in the *S. aureus* genome that are enriched with virulence genes and contain 26 out of 67 TA systems. We ectopically expressed multiple TA genes in *E. coli* and *S. aureus* that exhibited bacteriostatic and bactericidal effects on cell growth. The type I Fst toxin created holes in the cell wall while the TxpA toxin reduced cell size and induced cell wall septation. Besides, we identified a new TA system whose antitoxin functions as a transcriptional autoregulator while the toxin functions as an inhibitor of autoregulation. Altogether, this study provides a plethora of new as well as previously known TA systems that will revitalize the research on *S. aureus* TA systems.

## 1. Introduction

*Staphylococcus aureus* is a commensal pathogen that can cause a diverse array of infections and syndromes such as toxic shock syndrome, bacteremia, endocarditis, osteomyelitis, pneumonia, soft-tissue infection and many more [1]. *S. aureus* can detect and respond to diverse environmental stimuli such as nutrient starvation and stress to increase its fitness by altering the expression of numerous genes such as the toxin-antitoxin (TA) system and two-component system [2,3]. A typical TA system consists of two components: a toxin that can disrupt a cellular process and an antitoxin that functions as an antidote for the toxin. TA systems have been involved in virulence and persistent infections in *Escherchia coli* [4], *Mycobacterium* [5], and *Salmonella* [6]. They are classified into six types based on the mechanism of interaction between toxin and antitoxin [7]. The type I TA system has been characterized by an RNA-RNA interaction of the antitoxin antisense RNA with the toxin mRNA, while the type II TA system exhibits protein-protein interaction between toxin and antitoxin. In the type III TA system, the activity of the toxic protein is inhibited by the interaction with the antitoxin RNA, while toxin and antitoxin proteins in the type IV TA system compete for the same target [4,7,8]. The antitoxin protein in the type V TA system cleaves the toxin mRNA [9]. The newly identified type VI TA system differs from others in a way that the antitoxin functions as an adaptor to facilitate toxin proteolysis [10,11]. The abundance of TA loci has been considered to have evolved through horizontal gene transfer, by which TA systems successfully integrate into the bacterial genome and are involved in genetic stability as well as pathogenic potency [12]. Under unfavorable conditions such as starvation, bacteriophage attack, heat shock, and antibiotic stresses, the unstable antitoxin is degraded, and as a result, the toxin is freed to target vital cellular processes such as translation, transcription, and replication [7,10]. TA systems have been extensively studied in *E. coli* [4] and *Mycobacterium* [5], while the study on TA systems in *S. aureus* is less advanced. *S. aureus* possesses a number of TA genes in its genome, however just three type II and two type I TA systems have been studied [3]. The rest of TA systems still await discovery and analysis. Here, we have performed an extensive screening and assessment of TA loci in the genome of methicillin-resistant *S. aureus* MW2 utilizing available software and databases. We screened TA systems with Rasta bacteria [13], the toxin-antitoxin database (TADB) [14], and position-specific iterative basic local alignment search tool (PSI-BLAST) searches supported by the “guilt by association” principle and classified them into different families and types. HigBA was the most abundant TA family with 15 systems, followed by six GCN5-related acetyltransferases (GNATs) and five RelBE TA systems. Other TA families include MazEF, VapBC, HicBA, CcdBA, ParDE, HipBA, transporter TA systems, abortive infection (Abi) TA systems, and some with a domain of unknown function. Further, we ectopically expressed some toxin and antitoxin genes to assess their predicted functions. Most of the type II toxins did inhibit cell growth while their antitoxins did not show any inhibitory effects. We overexpressed RelE, MazF, and HigB toxins that exhibited growth inhibition. We detected seven type I TA systems including previously characterized type I toxins Fst and TxpA [15]. Transmission electron microscopy (TEM) analysis of the bacterial cell wall revealed that the Fst toxin broke the cell wall while TxpA and holin toxins increased cell wall septation. We also detected a new TA system and found that its toxin and antitoxin did not show any growth inhibition upon overexpression. In this new TA system, toxin and antitoxin can be co-transcribed, and the antitoxin can bind to the promoter and autoregulate its operon. The toxin protein interfered with the antitoxin function and inhibited binding of the antitoxin to the promoter. In summary, our present study has greatly expanded the plethora of TA systems in *S. aureus* and revealed a variety of genes involved in bacterial cellular processes.

## 2. Results and Discussion

### 2.1. Comprehensive Screening of Toxin-Antitoxin Systems in S. aureus

The typical toxin-antitoxin (TA) system consists of a stable toxin and an unstable antitoxin and is widely distributed in bacterial genomes. The toxin and antitoxin form a conjugative pair of genes that are co-transcribed, and are involved in various cellular processes, and importantly counterbalance each other. The fraction of the TA system encoded by a bacterial genome varies from one pathogen to another. *Mycobacterium* contains 88 [5], *E. coli* contains 40 [4], *Salmonella typhimurium* carries 27 [6,16], and *Pseudomonas aeruginosa* has 26 TA systems [17]. To date, three type II TA systems have been well characterized in *S. aureus*, namely Axe1-Txe1, Axe2-Txe2, and MazEF. These type II TA systems show similarity to *E. coli* Phd-Doc, YefM-YoeB, and MazEF systems, respectively [3].

**Figure 1:**
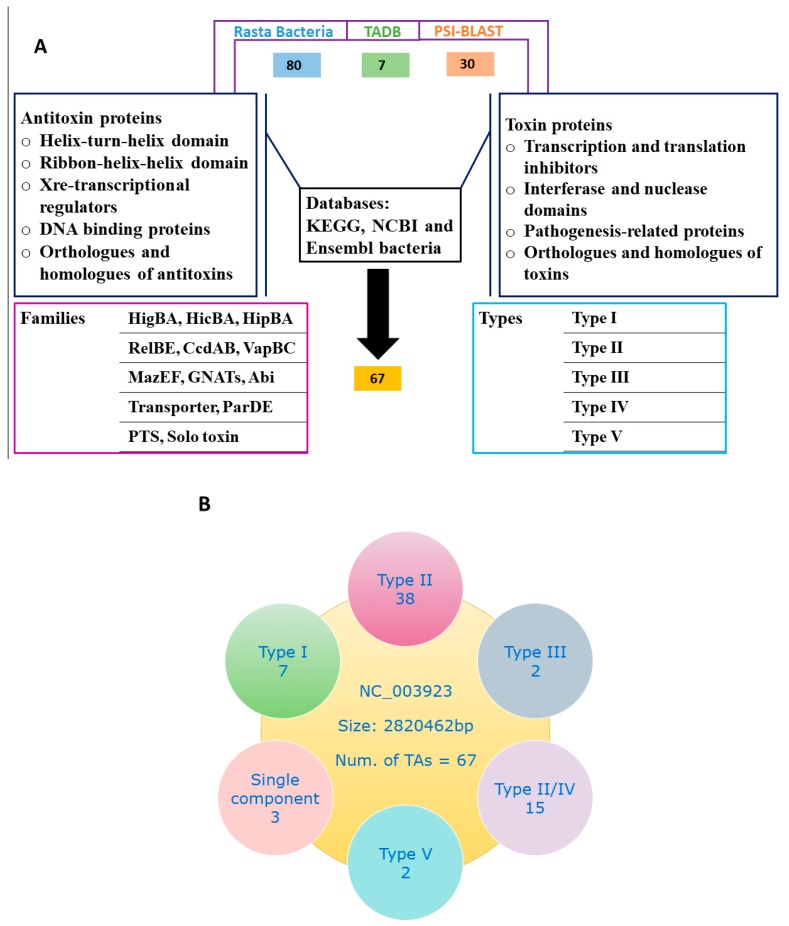
Screening of TA systems. (**A**) Preliminary screening of TA loci in *S. aureus* genome was performed with Rasta bacteria and toxin-antitoxin database (TADB) and followed by position-specific iterative basic local alignment search tool (PSI-BLAST) searches. In total, 80 putative TA systems are predicted by Rasta bacteria, seven by TADB, and 30 by PSI-BLAST. TA systems were manually curated by conserved domains such as helix-turn-helix (HTH), ribbon-helix-helix (RHH) and transcriptional regulators for antitoxin, and conserved domains such as interferases, nucleases, transcription and translation inhibitors and pathogenesis-related proteins for toxins. All selected TA system orthologues and homologs were analyzed, and finally, 67 putative TA systems were selected. (**B**) The selected 67 TA systems were classified into different families and types. Type II TA systems were abundantly detected followed by putative type II/IV TA systems.

In the present work, we combined multiple approaches and explored the *S. aureus* genome for putative TA loci. We screened for TA genes and predicted a total of 67 putative TA systems with Rasta bacteria, TADB, and PSI-BLAST. To make the classification more accurate, we used the TA systems of *E. coli* [4], *Klebsiella pneumoniae* [18], and *Mycobacterium* [5,19] as modal and manually curated each TA system and classified them into different families. We found type I, II, III, IV, and type V TA systems in *S. aureus* on the basis of software prediction, conserved domain analysis, and orthologues searches from Kyoto Encyclopedia of Genes and Genomes (KEGG). Details of the screening method and TA systems is given (Figure 1A,B), and the selected TA families are provided in Table 1.

### 2.2. Distribution of TA Genes in Different Families

**Figure 2:**
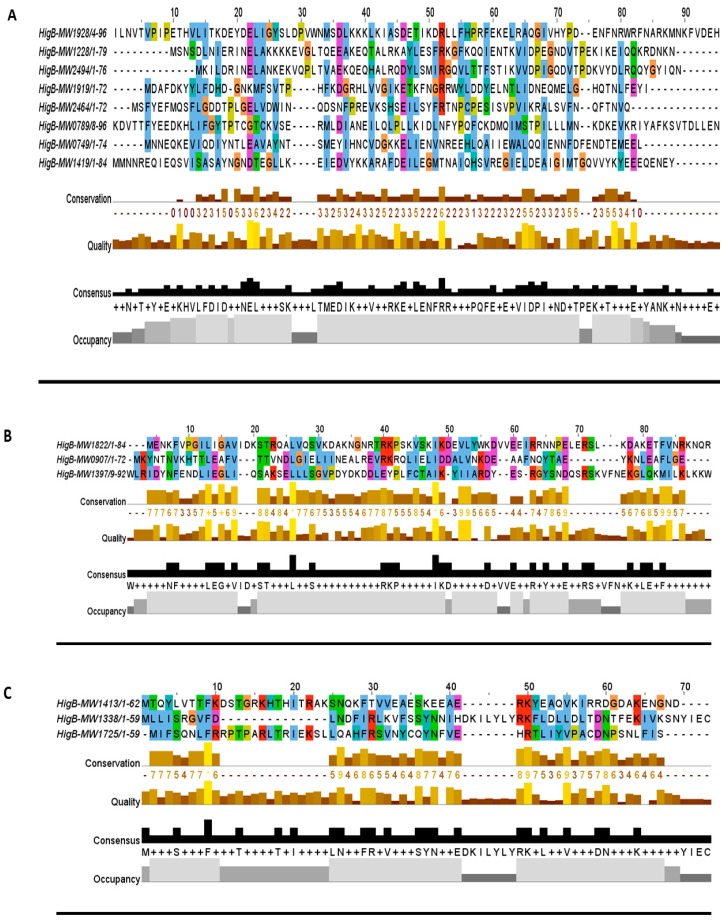
Multiple sequence alignment of HigB toxins. (**A**–**C**) The HigB sequences were aligned by MUSCLE software across the *S. aureus* genome. HigB toxins that were short in length and showed divergence in their family were aligned together in a sub-family as shown in (**B**,**C**).

The toxin gene codes for a poison protein and antitoxin is the cognate antidote. The toxin and antitoxin sequence domains are the determinants for families and TA types. The antitoxin mostly contains a DNA-binding domain such as helix-turn-helix (HTH) or ribbon-helix-helix (RHH) of the transcriptional regulator. For example, the RelBE family in *E. coli* is the most diverse family and most of the RelB antitoxins bind to the promoter region and autoregulate the TA operon [20]. In *Mycobacterium*, VapBC is the most abundant TA family, and the VapBC protein complex can autoregulate the TA operon [5]. When we screened *S. aureus* genome, we observed the enriched profusion of the host inhibition of growth (HigBA) TA family, which is a sub-family of RelBE super-family [14]. The HigBA TA system has the unique genetic annotation in type II TA systems, i.e., the toxin gene is upstream of the antitoxin, which makes it different from the rest of type II TA families [21,22]. HigB is a ribonuclease in function followed by HigA antitoxin, and they can form a heterotetrameric complex. The antitoxin contains an HTH-Xre domain and can bind to DNA, while the toxin targets 50S ribosomal subunit [22]. To date, no HigBA TA system has been reported in *S. aureus*. In this study, we identified 15 HigBA TA systems in the *S. aureus* genome and classified them into three groups by pairwise alignment to its close members (Figure 2A–C).

**Figure 3:**
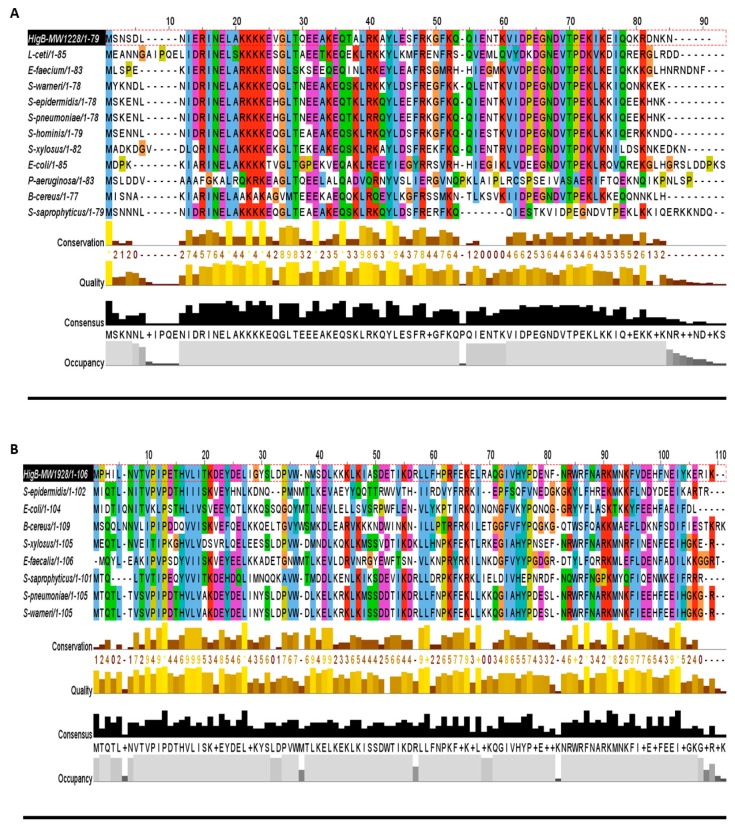

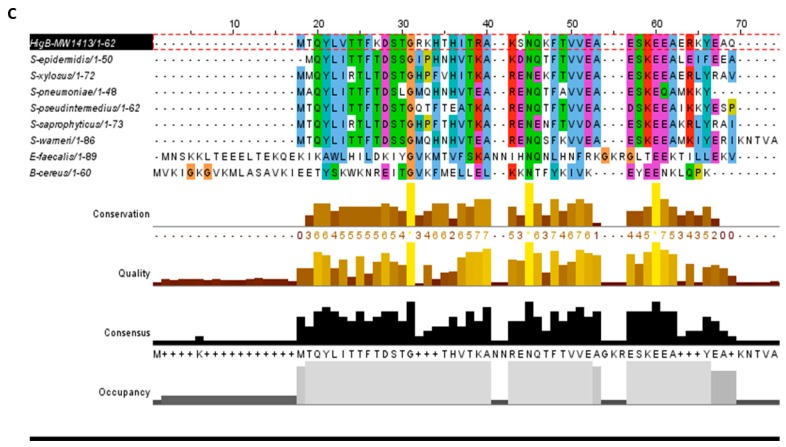
Homologs of the HigB toxins. (**A**) The HigB toxin MW1228 showed high conservation in *L. ceti*, *E. faecium*, *S. pneumoniae*, *E. coli*, *P. aeruginosa*, and *B. cereus*. The N-terminal 35 residues (13–48 aa) was the highly conserved region in all of the selected homologues. (**B**) The toxin MW1928 was the largest protein (106 aa) in the HigBA family and displayed a highly conserved region at N-terminal (10–27 aa) and a less conserved region at C-terminal (95–109 aa). (**C**) The toxin MW1413 is a small protein (62 aa) in the HigBA family and highly conserved in *Staphylococcus* species such as *S. epidermidius*, *S. xylosus*, *S. pseudointermedius*, *S. saprophyticus*, *S. warneri*, and *S. pneumoniae*. MW1413 was less conserved in *E. faecalis* and *B. cereus*.

In all three groups, the HigB toxin is followed by the HigA antitoxin, which satisfies the first requirement in predicating the HigBA TA system (Table 1). Such enriched abundance of HigBA TA systems has not been reported in other bacteria, but possible homologs can be detected. For example, homologues of HigB toxins (MW1228, MW1928, and MW1413) are prevalent in *Lactobacillus ceti*, *Enterococcus faecium*, *Streptococcus pneumoniae*, *E. coli*, *P. aeruginosa*, *Bacillus cereus*, and *Staphylococcus* species (Figure 3A–C). We aligned HigB toxin MW1228 with homologues from *L. ceti*, *E. faecium*, *S. pneumoniae*, *E. coli*, *P. aeruginosa*, and *B. cereus*, and detected a highly conserved domain of 35 residues (13–48 aa) at the N-terminus (Figure 3A). We did not detect a similar domain in other HigB toxins such as MW1928 and MW1413, but they also shared a unique domain with their homologues. When we aligned the homologues of MW1928 and MW1413, we detected a highly conserved domain of 17 residues (10–27 aa) in MW1928 and 11 residues (20–31 aa) in MW1413 homologues (Figure 3B,C). Although the homologues of the HigBA TA family are widely detected in many bacteria, mostly remain uncharacterized except in *Proteus vulgaris*, *Mycobacterium* [19], *Vibrio cholera* [23], and *P. aeruginosa*. In *P. aeruginosa*, the HigB toxin functions as RNase and reduces biofilm formation, pyocyanin and pyochelin production, and swarming motility [22]. The endonuclease toxin HigB cleaved AAA rich sequences as well as single monomer (A) in the coding region of *P. vulgaris* [24]. Interestingly, the HigB target coincided with amino acid lysine (AAA), one of the most frequent amino acid in the GC-less and AT-rich *Staphylococcus* species. Therefore, the enriched profusion of the HigBA system highlights the target specificity and functional sites in *S. aureus*.

**Figure 4:**
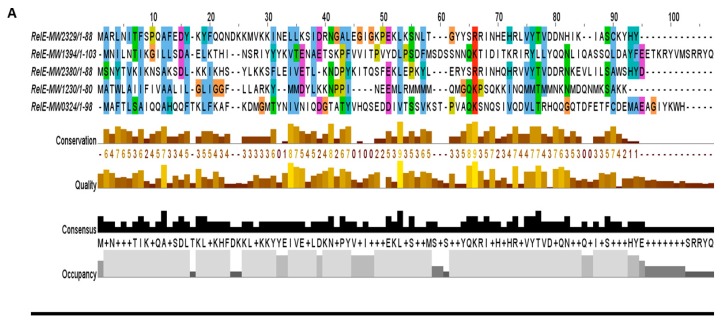

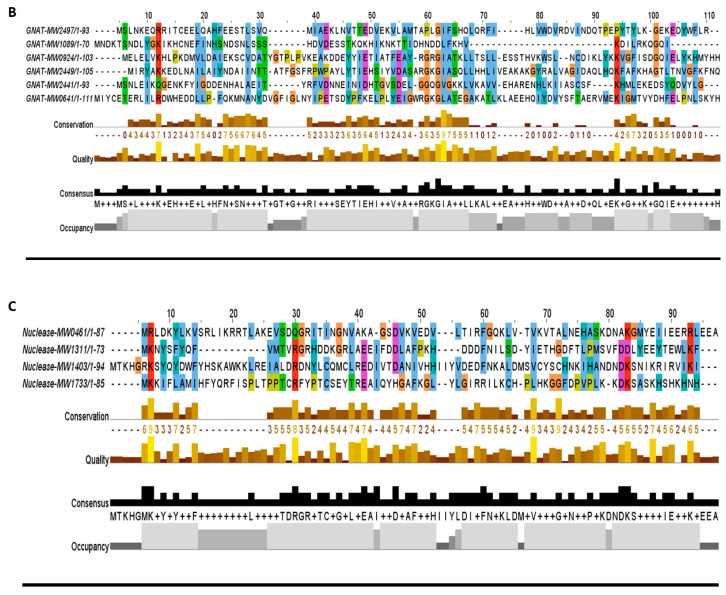
The predicted toxins were aligned by MUSCLE and sorted by pairwise alignment to close matching protein. (**A**) The RelE toxins MW2329, MW2380, and MW1230 were shorter in length and showed homology towards YefM-YoeB sub-family, while the MW0324 and MW1394 were longer in length and displayed less conservation, possibly making a new sub-family. (**B**) The GNATs toxins showed a functional site of 28 residues (41–68 aa) that contained the most conserved region in this family. (**C**) A moderate consensus site of 20 residues (30–49 aa) was detected in the nuclease family. The nuclease toxins MW1311, MW1403, and MW1733 were closely related in sequence alignment, while MW0461 was less conserved.

The RelBE super-family has sub-families, namely YefM-YoeB, MqsAR, PrlF-YhaV, and RelBE in *E. coli* [14]. The RelBE sub-family antitoxin RelB is degraded upon a stringent response that leads to the activation of the RelE toxin and inhibition of growth via targeting translation. Upon stringent response, RelB is degraded and subsequently leads to the activation of RelE, which targets translation and results in growth inhibition. The YefM-YoeB sub-family has been well characterized in *S. aureus* [3], *S. quorum* [25], S*. suis* [26], *Streptomyces* [27], *E. coli,* and *S. pneumoniae* [28]. The YoeB toxin can form a complex with the YefM antitoxin and target translation. In this study, we detected five RelBE TA systems (Figure 4A) that can be further classified into sub-families depending on their functions. For example, we characterized a new TA system of the RelBE super-family that showed resemblance with YefM-YoeB sub-family, and was involved in virulence control in *S. aureus* [20].

The GNATs-like TA system is the new addition of the TA family that can prevent peptide bond formation by acetylating tRNA and inhibiting translation in *Salmonella* and *Klebsilla* [18,29]. The TacT toxin inhibits translation via acetylating the initiator tRNA, and induces persister cell formation [29]. Six loci were detected in *S. aureus* that showed resemblance with GNATs TA systems (Figure 4B), and their toxins showed acetyltransferase-like domains.

Four TA loci showed resemblance with DNA gyrase inhibitors (nucleases) and were predicted to be either the ParDE or the CcdBA TA system (Figure 4C). The par toxin targets DNA gyrase and inhibits DNA replication in *E. coli* and *M. tuberculosis* [4,5]. The ParE-ParD components have been reported to form a heterotetrameric complex via the ParD N-terminal RHH domain that can increase the dimerization of ParD, while the C-terminal domain has a specific ParE toxin-binding site [30]. The CcdB toxin can bind to DNA gyrase, form a gyrase-DNA complex, and create a double-strand break into DNA. The CcdA antitoxin can resolve the complex by binding to the toxin protein and dislodge CcdB from gyrase [31]. So far, these loci have not been studied in detail in *S. aureus* and characterization of these systems will reveal the mechanism of toxin action.

The first persistence-associated gene *hipA* was first detected in 1983 [32] and later studied in *E. coli* as the HipBA TA system [33]. We found one HipBA-like system in *S. aureus* that is different in sequence and length, but its orthologue search revealed its relatedness with the HipBA family. Here we named it as the first HipBA TA system in *S. aureus*. In the *hip* operon, HipA is a toxin that phosphorylates glutamyl-tRNA synthetase, while HipB is the antitoxin that counteracts the HipA toxicity [33].

The VapBC TA system is the most abundant TA system in prokaryotes and is found copiously in *E. coli*, *Mycobacterium*, *Shigella*, *Sulfolobus*, and *Leptospira* [2,4,5,34], while there is no report of *vapBC* loci in *S. aureus*. The toxin VapC targets translation by cleaving mRNA or tRNA. The VapB antitoxin is usually a transcriptional regulator that has a DNA binding domain and forms a complex with VapC. VapBC can respond to different stress conditions such as hypoxia, antibiotics stress, and latent infections [34,35]. Collectively, we detected 3 VapBC TA systems in *S. aureus* (Table 1), which have not been characterized so far.

**Figure 5:**
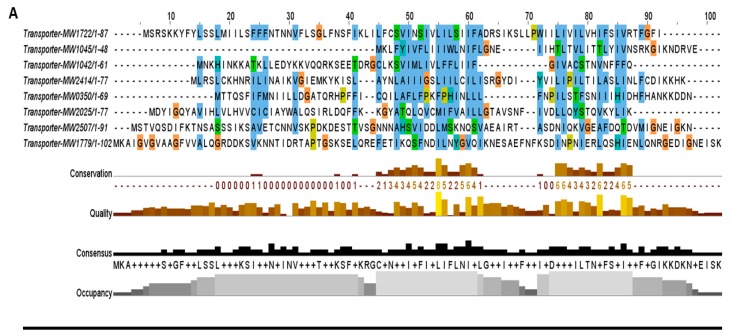

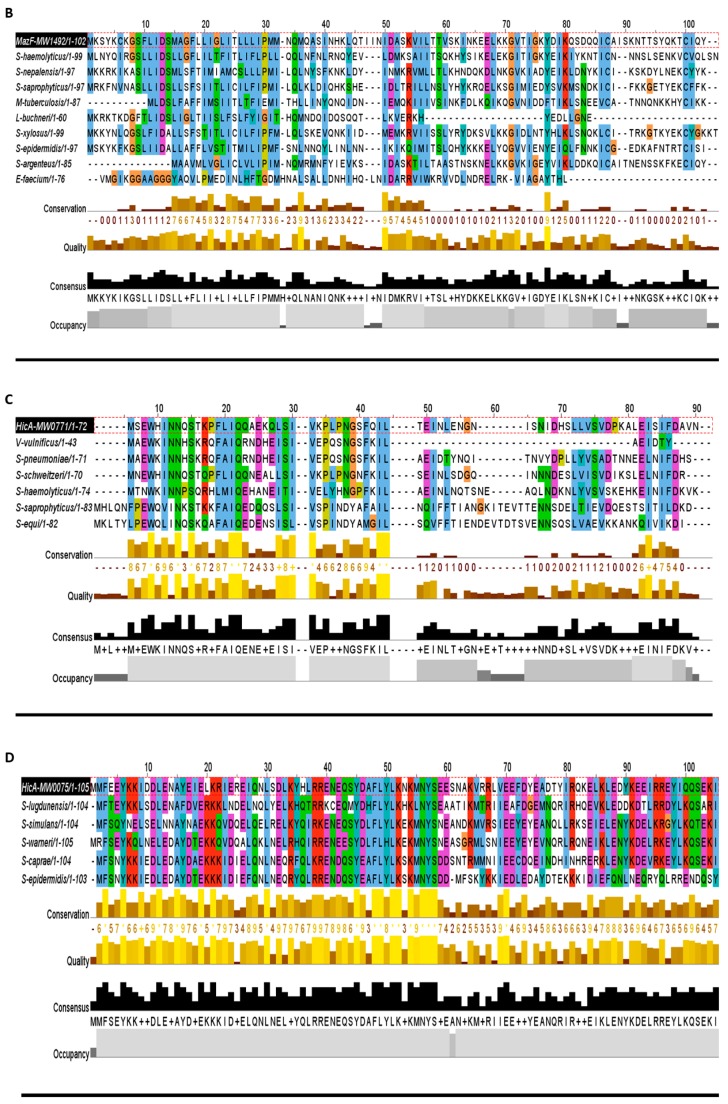

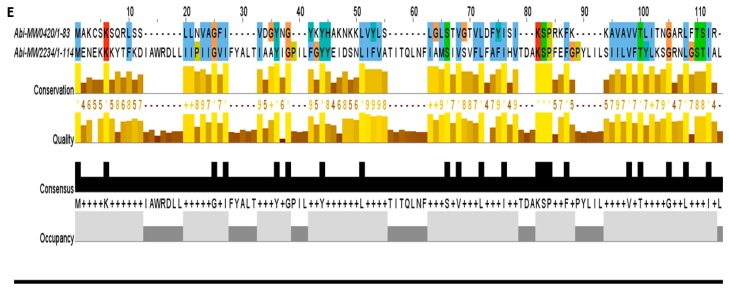
The toxins were aligned by MUSCLE and sorted by pairwise alignment to close matching protein. (**A**) The transporter family toxins displayed less conservation among all TA families because each of the protein is associated with different secretion systems and transport pathways. The 15 residues (48–62 aa) are the highly consensus sites in this family. (**B**) Homologs of MazF toxin MW1492 were aligned that showed a high degree of conservation in *M. tuberculosis*, *L. buchneri*, *E. faecium*, and *Staphylococcus* species. The highly conserved region contains 25 residues (8–32 aa) at N-terminus. (**C**) The HicA toxin MW0771 homologs were detected in *Vibrio vulnificus*, *S. pneumoniae*, and *Staphylococcus* species. The 20 residues (11–30 aa) are the highly consensus sites in the homologs of MW00771 protein. (**D**) The homologs of MW0075 could not be detected in *M. tuberculosis*, *L. buchneri*, *E. faecium*, *V*. *vulnificus*, *S. pneumoniae* and *B. cereus*. The MW0075 toxin is a highly conserved protein in *Staphylococcus* species. (**E**) The two Abi toxins were aligned by pairwise alignment that showed 16% sequence similarity. Both Abi toxin homologs could be detected in different bacterial species.

The second most abundant family that consists of nine TA systems belongs to the transporter TA family in *S. aureus*. These TA systems are either located in secretion systems or contain conserved domains associated with transport pathways. For example, toxin MW0268 and antitoxin MW0269 are located in the type VII secretion system while toxin MW2507 is an effector protein that showed proximity towards the type VII secretion system secretory proteins EsxA and EsxB. Similarly, toxins MW1042 and MW1722 are associated with ATP-binding cassette transporter permeases, while toxin MW2414 has orthologue homology with TerC protein that has been implicated in the efflux of tellurium ions in *E. coli* [36]. The toxins MW1779 and MW0350 are related to sodium and hydrogen antiporter systems. These putative TA systems were grouped into the transporter TA family, and their toxin proteins were aligned together that were less conserved and more divergent (Figure 5A). We believed that these systems might be involved in inhibition and regulation of secretion systems and transport pathways.

The MazEF family has been characterized by the toxin MazF that acts as an endoribonuclease targeting mRNA, 16S and 23S ribosomal RNAs in *Mycobacterium* and *E. coli* [5,10]. Components of the MazEF TA system are commonly called global translation inhibitors [37], and are activated upon nutrient or oxidative stress, DNA damaging response, and heat shock [5]. The MazEF TA systems have been studied in *E. coli*, *M. tuberculosis*, *Clostridium difficile*, and *S. aureus* [2,3,4,5]. In the present study, we detected one new and one previously studied MazEF systems. The new MazF toxin showed a high degree of conservation in other bacteria including *E. faecium*, *M. tuberculosis*, and *lactobacillus buchneri* (Figure 5B).

When we mined the *S. aureus* genome, we discovered two HicBA TA systems (Figure 5C,D). In the HicBA TA system, *hicA* codes for the toxin that cleaves mRNA and reduces the rate of translation, while *hicB* rescues the bacteriostatic effects induced by *hicA* [38]. The transcription of *hicBA* is induced upon carbon and amino acid starvation and depends on lon protease in *E. coli* [39].

Apart from the type II TA system, two loci were predicted as the abortive infection system (Abi) TA system that can probably constitute the type III or IV TA system (Figure 5E). The TA system ToxIN in *Pectobacterium atrosepticum* was the first TA system described that also acted as the abortive infection system. This system can lead to altruistic cell death and viral replication inhibition [40,41]. The Abi TA system has not been discovered in *S. aureus* so far and awaits further investigation

**Figure 6:**
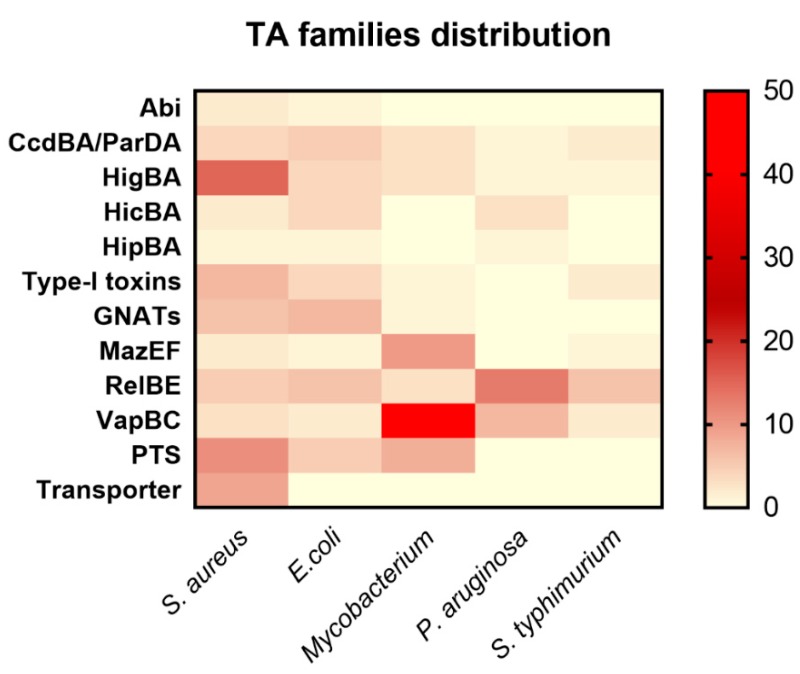
Distribution of TA families in *S. aureus*, *E. coli*, *Mycobacterium*, *P. aruginosa,* and *S. typhimurium*. The selected TA families were compared with the reported TA families of other bacteria. The highest number of TA systems in *Mycobacterium* is 50 VapBC, 10 MazEF, 8 PTS, and 3 HigBA TA systems. *P. aruginosa* has 13 RelBE, 7 VapBC, and 3 HicBA systems. *E. coli* has 7 GNATs, 6 RelBE, 5 nuclease toxins, and 4 each HigBA and HicBA systems. *S. typhimurium* has 6 RelBE, 2 VapBC, and 2 nuclease toxins. Seven type I toxins were found in *S. aureus*, 4 in *E. coli*, 2 in *S. typhimurium*, and 1 in *Mycobacterium*. The HipBA TA system was the rarest family that has not been discovered in any bacteria except *E. coli*, *P. aeruginosa,* and *S. aureus*.

The *S. aureus* genome contains 11 TA systems that neither show any similarity with the reported TA families nor contain conserved domain. Therefore, we kept them as putative TA systems (PTS). Among these PTS, 3 TA systems can be three components (MW1430-1431-1432, MW1149-1150-1151, and MW0776-0777-0778) or two components including toxin and antitoxin as shown in Table 1. The third component can be either a second antitoxin or transcriptional regulator that probably alters the expression of TA genes. Three of the PTS were classified as a solo toxin (MW0101, MW0036, and MW1827) that can probably constitute different types of TA systems or might function differently (Table 1). In general, three-component TA systems and some solo toxins have been studied in detail in cyanobacteria [42]. Further research on these PTS systems may reveal their classification and function in *S. aureus*. In conclusion, we compared *S. aureus* TA families with the reported TA families of *E. coli*, *Mycobacterium*, *P. aeruginosa*, and *S. typhimurium*, and our results could advance our understanding of the distribution of TA families in bacteria (Figure 6).

### 2.3. Pathogenicity Islands TA Systems

Pathogenicity islands (PIs) are unique regions of DNA and consist of virulence genes and mobile genetic elements. We detected 7 PIs in *S. aureus* genomes by using PIs database [12]. These islands are enriched with TA-like genes. The roles of TA systems in PIs include but are not limited to successful integration, maintenance and stability of islands, and defense against phages [12,43]. The PI phi-Sa-2 and VSa-gamma have 11 and 4 TA systems (Table 2). We predicted that PIs-TA systems are the most functional TA systems in *S. aureus*. This prediction is supported by previous reports that mechanistically related TA systems can display different functions depending on their localization in regions such as PIs [43]. In total, we detected 26 TA systems out of 67 that are located within pathogenicity island (PI) regions with an abundance of virulence genes in the neighborhood.

### 2.4. Functional Assessment of the Type I TA System

**Figure 7:**
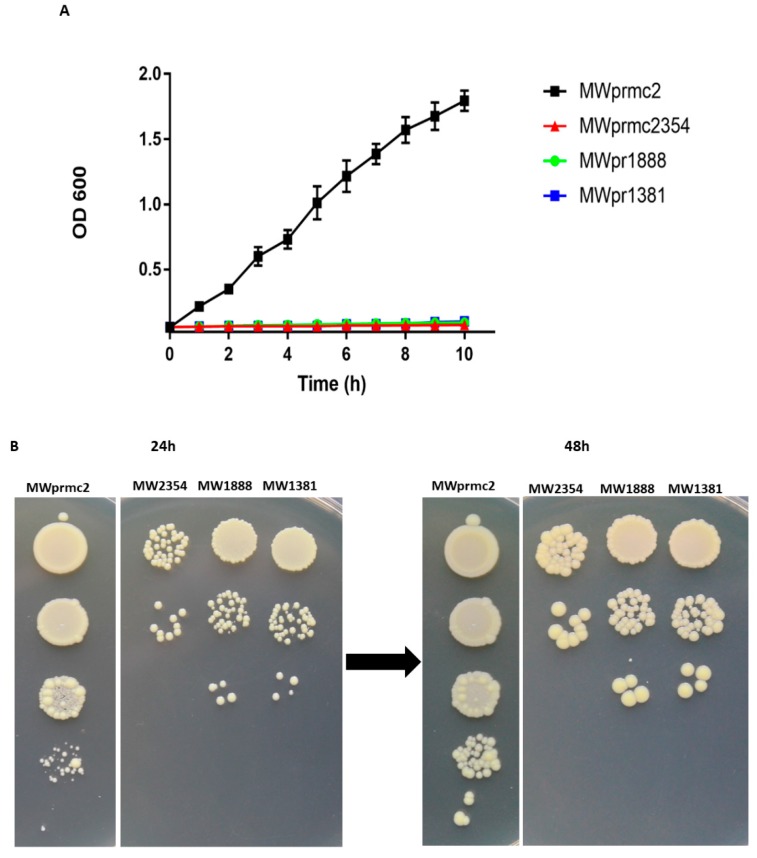
Overexpression of type I toxins. (**A**) Normal growth was observed in MWprmc2 that contains an empty pRMC2 vector while complete growth inhibition was observed in MWprmc2354, MWprmc1888 and MWprmc1381 that contain Fst, TxpA, and holin toxins, respectively. (**B**) The bactericidal nature of three type I toxins was determined by ATC inducible plates, and the results showed that cells expressing Fst, TxpA, and holin toxins were unable to restore growth as compared to control MWprmc2.

The type I TA system toxins are called bacterial time bombs because these toxins target the bacterial cell wall and induce cell death. Importantly, type I toxins are considered to be the most critical peptides to eradicate *S. aureus* cells [3,44]. Here we have detected seven type I TA systems that have toxin domains such as TxpA, Fst, and holin (Table 1). We have also revealed two type I TA systems Fst and TxpA that were previously reported [3,15]. To assess the activity of three type I toxins that target cell wall, we constructed different recombinant vectors as shown in Materials and Methods. The toxin was ectopically overexpressed in *S. aureus* and the OD_600_ value was determined. The expression of toxins inhibited cell growth, confirming the deadly nature of type I toxins (Figure 7A). Further, the cellular toxicity assay was performed on anhydrotetracycline (ATC) inducible plates. The results indicated that cell growth was inhibited and cannot be restored (Figure 7B), demonstrating that type I toxins are bactericidal and can induce cell death in *S. aureus*.

### 2.5. Transmission Eelectron Microscopy Analysis of the Bacterial Cell Wall

**Figure 8:**
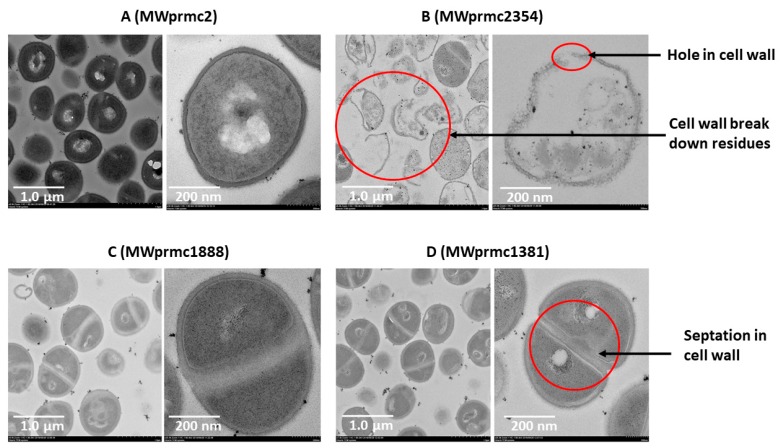
The bacterial cell wall was analyzed by TEM. Single cell micrographs were captured at magnification ×25.0 K and scale bar 200 nm while the group of cells micrographs were captured at magnification ×8.0 K and scale bar 1.0 µm. (**A**) Mwprmc2 was an empty vector and used as control. (**B**) The Fst toxin (MWprmc2354) broke down the cell wall as well as created holes. (**C**,**D**) The TxpA (MWprmc1888) and holin (MWprmc1381) toxins increased cell wall septation and reduced cell size compared to the control MWprmc2 with the empty vector.

Type I toxins are short peptides and apoptotic in nature and target cell wall [45]. We ectopically expressed three type I toxins for 1 h, harvested the cells, and analyzed the cell wall by transmission electron microscope (TEM). As a control, the *S. aureus* with empty pRMC2 vector was used (Figure 8A). The type I toxins targeted the cell wall differently. The MWprmc2354 (Fst toxin) created holes and broke the cell wall, and its lysis activity was intense. Apart from this, empty cells with no cellular entities were observed, and cells unable to withstand the toxin effects and ultimately cell wall remaining remnants were detected with ectopic expression of Fst (Figure 8B). The ectopic expression of MWprmc1888 (TxpA toxin) and MWprmc1381 (holin toxin) led to neither holes nor breakage, and instead increased wall septation. Two cells intersecting their cell walls and septum formation were ostensible with the TxpA (Figure 8C) and holin (Figure 8D) toxins as shown by TEM micrographs (Figure 8A–D).

### 2.6. Response and Function of Cellular Proteases in S. aureus

**Figure 9:**
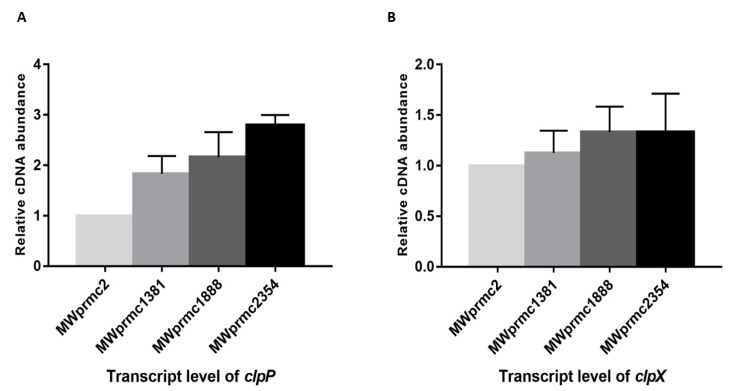
The transcript levels of *clpP* and *clpX* were determined by quantitative real-time PCR (qRT-PCR). The transcript levels were compared with MWprmc2 containing empty pRMC2 vector. (**A**) The ectopic expression of MWprmc2354 toxin activated *clpP* (*p* ≤ 0.06) when compared to other type I toxins MWprmc1888 and MWprmc1381 (*p* ≤ 0.08). (**B**) The ectopic expression of type I toxins did not alter the transcript level of *clpX*.

Most of TA systems contain a liable antitoxin that is either degraded by cellular proteases or suppressed to enhance toxin expression under stress conditions, suggesting that the antitoxin can be a positive or a negative regulator of the toxin. We detected six different types of Clp proteases; ClpA, ClpB, ClpC, ClpP, ClpQ, and ClpX in *S. aureus.* Among them, ClpC and ClpX form complexes with ClpP and are involved in the protein homeostasis by either conditional degradation or disposing of the unwanted proteins [46]. In the present study, we observed the upregulation of *clpP* towards type I toxins, while *clpX* remained steady (Figure 9A,B). Our data indicated that *clpP* was activated when the toxin was ectopically expressed. This result suggests that the proteases play an essential role in keeping the toxin level in the balanced state in order not to severely damage the bacterial cells. The response and function of proteases towards TA systems are an exciting topic and need further study.

### 2.7. Functional Assessment of Type II TA Systems

**Figure 10:**
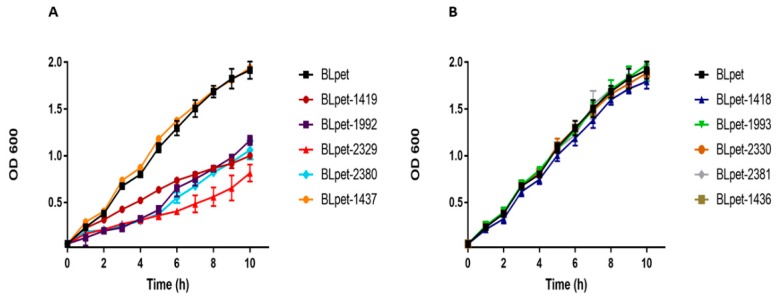
Expression of type II toxins and antitoxins in *E. coli* BL21. (**A**) The ectopic expression of RelE toxins (BLpet-2329 and BLpet-2380), HigB toxin (BLpet-1419), and MazF toxin (BLpet-1992) inhibited cell growth while BLpet-1437 has no inhibitory effects on cell growth. (**B**) RelB antitoxins (BLpet-2330 and BLpet-2381), HigA antitoxin (BLpet-1418), MazE antitoxin (BLpet-1993), and BLpet-1436 did not inhibit cell growth.

The type II TA system is well studied in *E. coli*, *Mycobacterium*, *Pseudomonas*, and *Staphylococcus*. After screening the TA loci in *S. aureus*, we randomly selected five type II TA systems from different families, i.e., MW2329-MW2330 and MW2380-MW2381 TA systems from the RelBE family, MW1992-MW1993 TA system from the MazEF family, MW1419-MW1418 TA system from the HigBA family, and MW1436-MW1437 TA system from the PTS family. We aimed to confirm whether the predicted toxin or antitoxin can inhibit cell growth or not. We ectopically expressed toxins and antitoxins in *E. coli* BL21 and monitored the bacterial growth. All the toxins inhibited cell growth except MW1437, while all the antitoxins were similar to control BLpet with empty pET28a plasmid (Figure 10A,B). We conclude that most of the type II TA system toxins target metabolic pathways and induce bacteriostatic conditions, contrasting to type I toxins with bactericidal activities.

**Figure 11:**
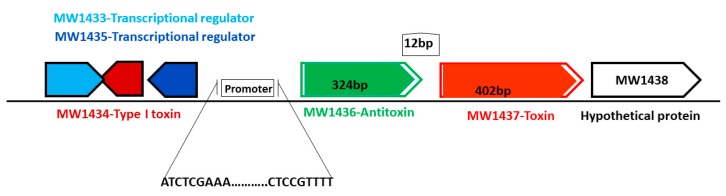
Schematic representation of a new TA system from phi-Sa-2 pathogenicity island. The toxin-coding gene is 402 bp, the antitoxin-coding gene is 324 bp in length, and they are located close to the type I TA system and transcriptional regulator.

The MW1436-MW1437 TA locus was predicted to be either a type II or a type IV TA system and classified as PTS (Table 1). Locus MW1437 codes for a toxin while MW1436 codes for an antitoxin. The two genes are separated by a 12 bp intergenic region and are located in the phi-Sa-2 PI region within the neighborhood of one of the type I TA system (MW1434) (Figure 11). The toxin protein has a Zn-dependent protease-like domain, while the antitoxin is an HTH domain-containing transcriptional regulator. The ectopic expression of both toxin BLpet-1437 and antitoxin BLpet-1436 were growth-supportive, and no cellular toxicity was observed (Figure 10A,B).

**Figure 12:**
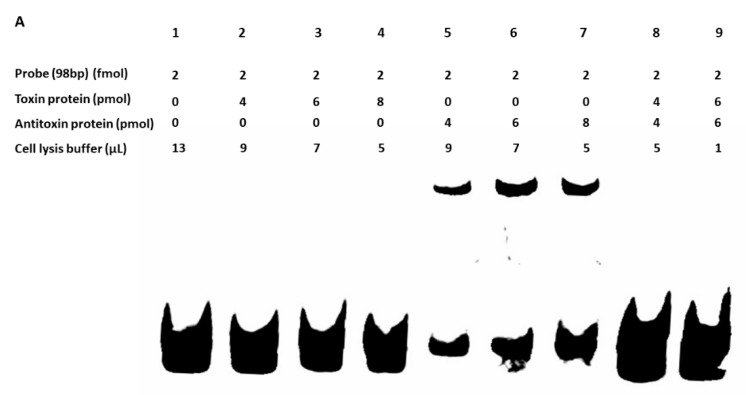

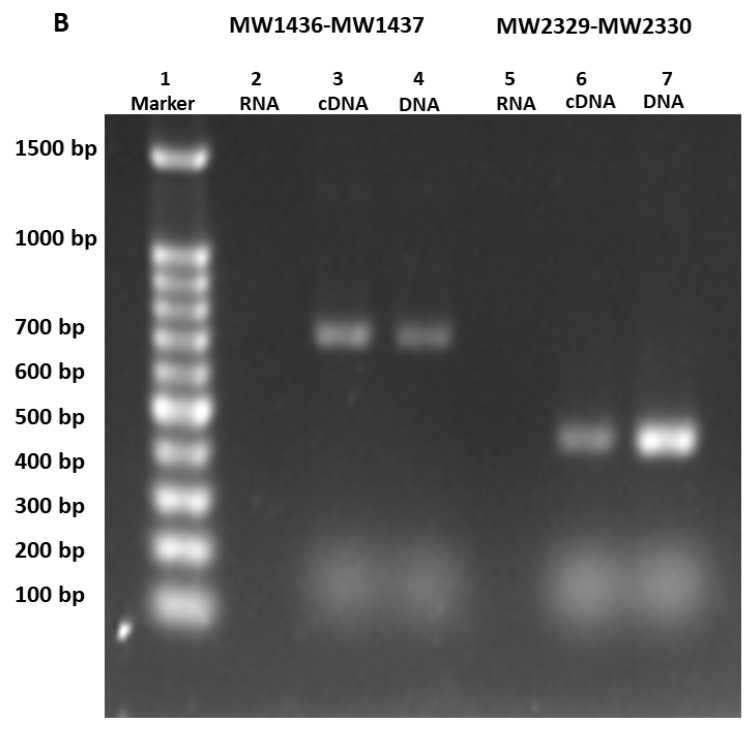
(**A**) Gel-shift assay was performed to detect the binding of toxin and antitoxin to the promoter. The up-shift represents the binding of the antitoxin protein to the 98 bp probe (lanes 5–7), while the toxin protein was unable to bind (lanes 2–4). No shift was observed when antitoxin and toxin proteins were mixed together with the 98 bp probe (lanes 8 and 9). (**B**) The TA genes MW1436-MW1437, and MW2329-M2330 were co-transcribed. 1 = marker, 2, 5 = RNA, 3, 6 = cDNA, and 4, 7 = DNA. The co-transcript product (lanes 3 and 4) represents the 700 bp size of MW1436-MW1437, and lanes 6 and 7 represent the 400 bp size of MW2329-MW2330.

We did not detect the in vivo protein–protein interaction between toxin (BLpet-1437) and antitoxin (BLpet-1436) proteins (data not shown). Further, we evaluated the DNA-binding ability of antitoxin and toxin proteins. Firstly, we determined the 98 bp promoter region of this operon using SoftBerry software. We incubated the toxin and antitoxin separately as well as together with the 98 bp biotin-labeled promoter probe. Our gel-shift assay results indicated that the antitoxin can bind to the probe and probably autoregulate this operon (Figure 12A, lanes 5–7), while the toxin could not bind to the probe (Figure 12A, lanes 2–4). Interestingly, the addition of the toxin protein to antitoxin and the probe mixture inhibited antitoxin–probe complex formation, suggesting that the toxin protein can inhibit the binding of antitoxin to the promoter region and prevent autoregulation (Figure 12A, lanes 8 and 9). To further determine whether the two genes can be co-transcribed, we performed RT-PCR using RNA, cDNA, and DNA as templates and detected the 700 bp co-transcript of MW1436-MW1437 (Figure 12B, lanes 3 and 4). Our data also showed that *relBE* genes MW2329-MW2330 were co-transcribed (Figure 12B, lanes 6 and 7). In summary, although the toxin of this TA system did not inhibit cell growth, it may be involved in some other functions such as autoregulation of the neighbor genes or stability of the phi-Sa-2 island.

## 3. Conclusions

We comprehensively screened all the possible TA loci in *S. aureus* genome and predicted previously unidentified 67 TA systems, and classified them into different families and types. Our data substantially expanded the collection of TA systems in *S. aureus* and revealed the nature of type I and type II toxins. We detected a new TA system whose toxin did not inhibit cell growth but prevented cognate antitoxin binding to the promoter. The presence of these chromosomally encoded TA systems suggests that these loci may be involved in *S. aureus* physiology and genome stability. More importantly, our prediction may open a new window for further study of these uncharacterized TA systems, especially, their association with *S. aureus* pathogenicity and virulence.

## 4. Materials and Methods

### 4.1. Bacterial Strains and Growth Conditions

The bacterial strains used in this study are listed in Table S1 of the Supplementary Material. S. aureus was grown in tryptic soy broth (TSB) medium and E. coli was grown in lysogeny broth (LB). pRMC-2, an ATC inducible shuttle vector, and pET28a, an isopropyl β-D-1-thiogalactopyranoside (IPTG) inducible plasmid, were used in S. aureus and E. coli BL21, respectively. For plasmid maintenance, each medium was supplemented with appropriate antibiotic (ampicillin 150 µg/mL, chloramphenicol 15 µg/mL, and kanamycin 50 µg/mL). Cells were washed with normal saline. Normal growth conditions for S. aureus and E. coli were 37 °C and 220 rpm. Growth curves were determined by measuring optical density at 600 nm using a BioTek microplate reader.

### 4.2. Screening of TA Systems in S. aureus

Initially, we identified all putative TA genes in *S. aureus* by Rasta bacteria and TADB. Rasta bacteria cutoff score >53 was set and 80 TA systems were selected, and seven TA systems were predicted by TADB. Further, we performed PSI-BLAST searches using *Mycobacterium* and *E. coli* known TA systems as modal and selected 30 more TA systems in *S. aureus*. To improve the screening strategy, we analyze the predicted TA domains in databases such as KEGG, NCBI, and Ensembl bacteria, and analyzed the orthologues and homologs. Then the selected TA systems were classified into different families and types. The approach is summarized in Figure 1A.

### 4.3. Construction of Vectors

TA genes were amplified by PCR and inserted into pRMC2 vectors via KpnI-SacI-EcoRI and pET28a via NdeI-BamHI-EcoRI-XhoI restriction enzymes, resulting in the recombinant vectors MWprmc2354, MWprmc1888, MWprmc1381, BLpet2329, BLpet2330, BLpet2380, BLpet2381, BLpet1992, BLpet1993, BLpet1418, and BLpet1419. MWprmc2 and BLpet were empty vectors and used as controls in *S. aureus* and *E. coli,* respectively. All the constructed vectors were confirmed by DNA sequencing. The primers used for the construction of vectors are provided in Table S1 of the Supplementary Material.

### 4.4. Cellular Toxicity Assay

The constructed vectors were transformed into *E. coli* Trans T1 and BL21 by heat shock at 42 °C for 1 min. The pRMC2-vectors were initially transformed into *E. coli* Trans T1 by heat shock and then to *S. aureus* RN4220 and *S. aureus* MW2 by electroporation. Further, the 24 h culture was diluted 200 times and grown at 37 °C for 2 h. When the culture reached OD_600_ = 0.45–0.48, ATC (200 ng/mL) or IPTG (0.1 mM) was added, and the growth pattern was monitored for 10 h at 1 h intervals. The bactericidal nature of type I toxins was assessed on ATC plates. *S. aureus* was grown as described above to OD_600_ = 0.85–0.90. Cells (normally 5 × 10^8^ CFU/mL) were serially diluted in water, and 10 µL from each dilution was spotted on ATC inducible plates. The plates were incubated at 37 °C and observed for the restoration of growth after 24 h and 48 h intervals.

### 4.5. RNA Extraction and qRT-PCR

*S. aureus* was grown in TSB medium under specified conditions such as ATC induction for 1 h. Then, 1 mL of culture was centrifuged at 12,000 rpm for 2 min and the cells were washed and suspended in RNAiso Plus. Cells were treated with 0.1 mm silica beads in RNA isolation tube by the FastPrep-24 Automated system for 40 s twice. Trichloromethane was added in the RNA isolation tube and kept at 4 °C for 5 min, and then centrifuged at 12,000 rpm at 4 °C for 20 min. The up-layer was taken and mixed with an equal volume of isopropanol and kept at −20 °C for 2 h. Then the tubes were centrifuged at 12,000 rpm for 30 min, washed with 75% ethanol, air dried and dissolved in water (50 µL). RNase-free DNase I was used for residual DNA removal, and then RNAiso Plus and trichloromethane were added and centrifuged at 12,000 rpm at 4 °C for 20 min. The up-layer supernatant was transferred to a new tube and mixed with an equal volume of isopropanol and kept at −20 °C for 2 h. The tubes were centrifuged at 12,000 rpm at 4 °C for 30 min, and RNA was collected and washed with 75% ethanol and air dried. RNA concentration and quality were determined by Beckman Coulter and NanoDrop. The PrimeScript First Strand cDNA synthesis kit and SYBR Premix Ex Taq kit (TaKaRa) were used for RT and real-time PCR, respectively. The gene signal was normalized with *gyrA* or *hu* cDNA abundance [47]. All the qRT-PCR primers were designed by Beacon designer 7 and synthesized by General Biosystems China and listed in Table S1 of the Supplementary Material.

### 4.6. Preparation of Samples for Transmission Electron Microscopy

The type I toxins were ectopically expressed for 1 h in *S. aureus*, and cells were harvested and washed with phosphate buffered saline (PBS). The samples were then fixed with 2% paraformaldehyde and 2.5% glutaraldehyde in PBS buffer at 4 °C overnight, and washed three times in PBS buffer, then fixed with 2% Osmium tetroxide in PBS buffer at 4 °C overnight, and washed three times in PBS buffer. After fixation, the samples were dehydrated by a graded series of ethanol (30%, 50%, 70%, 80%, 90%, 95%, and 100%) for 20 min at each step, and transferred to absolute acetone for 20 min. The samples were placed in 1:1 mixture of Spurr resin and absolute acetone at room temperature for 1 h, and then transferred to 3:1 mixture of Spurr resin and absolute acetone at room temperature for 3 h, and to Spurr resin mixture overnight. Then the samples were placed in capsules contained embedding medium and heated at 70 °C overnight. The specimen sections were stained by uranyl acetate and alkaline lead citrate for 5–10 min, respectively, and images were captured with a Hitachi HT-7700 transmission electron microscope.

### 4.7. Gel-Shift Assay

Toxin and antitoxin expression vectors BLpet1437 and BLpet1436 were transferred into *E. coli* BL21 cells, and the transformants were grown in LB medium at 37 °C to OD_600_ = 0.45–0.48, and induced with IPTG (0.1 mM) at 30 °C for 5 h. Kanamycin 50 µg/mL was added into LB medium to maintain the plasmid. Cells were harvested, washed with normal saline, and lysed by sonication for 15 min in lysis buffer (50 mM Tris-HCl, pH 8.0, 300 mM NaCl). The proteins were purified by Ni^2+^-NTA histidine affinity column and concentrated by Millipore protein concentration column. Then, the biotin label primer was synthesized and the 98 bp promoter region was amplified by PCR and incubated with toxin and antitoxin proteins at 37 °C for 1 h. The 15µL reaction mixture contains probe (2 fmol), each toxin and antitoxin protein (4–8 pmol), and lysis buffer. The reaction was stopped by adding gel-shift loading dye and heated at 95 °C for 10 min, and then loaded on 4% native polyacrylamide gel and run in 0.5× Tris-borate-EDTA buffer at 400 mA for 30 min as previously described [20]. DNA bands were transferred onto nylon membrane and detection was performed with chemiluminescent detection kit. Image Quant LAS 4000 mini imaging system was used to detect the membrane.

### 4.8. Statistical Analysis

The nucleotides and protein sequences were obtained from the NCBI database and analyzed by Vector NTI software. For protein sequence alignment, MUSCLE [48] software was used and visualized by Jalview. Graph pad prism and Origin pro 9 was used for data analysis. Primers were designed by Beacon designer 7. Welch *t*-test was used for statistical significance.

## Figures and Tables

**Table 1:** List of all selected TA systems and their predicted families. The TA systems were arranged in order of their proximity to the TA family. The length of toxin and antitoxin proteins is given in amino acids (aa).

**Family**	**Toxin Genes**	**Length (aa)**
TxpA/Fst/Holin (type I toxins)	MW1888-TxpA	44
	MW2354-Fst	84
	MW0911-TxpA	35
	MW1440-TxpA	34
	MW0405	64
	MW1434	81
	MW1381-Holin	100
**Family**	**Toxin/Antitoxin Genes**	**Length (aa)** **Toxin/Anti-toxin**
HigBA (type II)	MW1419/MW1418	84/57
	MW1725/MW1724	59/117
	MW2494/MW2493	76/106
	MW1228/MW1227	79/77
	MW1413/MW1412	62/66
	MW0749/MW0748	74/48
	MW1338/MW1337	59/116
	MW1928/MW1927	106/53
	MW1397/MW1396	92/110
	MW1822/MW1821	91/154
	MW0789/MW0788	98/128
	MW1919/MW1918	72/134
	MW2464/MW2463	72/275
	MW0907/MW0906	72/189
	MW1056/MW1055	44/62
RelBE (type II)	MW2329/MW2330	88/83
	MW2380/MW2381	88/85
	MW1230/MW1231	80/155
	MW0324/MW0325	129/67
	MW1394/MW1395	131/133
GNATs (type II)	MW2441/MW2440	94/144
	MW1089/MW1088	70/203
	MW0641/MW0640	180/99
	MW0924/MW0923	160/71
	MW2449/MW2450	163/56
	MW2497/MW2498	99/185
Nucleases (type II) (CcdAB/ParDE)	MW1311/MW1312	73/166
	MW0461/MW0462	87/130
	MW1403/MW1402	104/101
	MW1733/MW1732	85/159
VapBC (type II)	MW1070/MW1071	263/224
	MW0302/MW0303	266/315
	MW0572/MW0573	211/295
MazEF (type II)	MW1992/MW1993	120/56
	MW1492/MW1493	114/144
HicBA (type II)	MW0075/MW0076	109/83
	MW0771/MW0772	72/94
Transporters (type II/IV)	MW1042/MW1043	61/77
	MW1045/MW1046	48/65
	MW2414/MW2413	77/102
	MW1722/MW1723	100/147
	MW2025/MW2026	77/134
	MW1779/MW1780	121/68
	MW0350/MW0349	69/83
Transporters (type V/II)	MW2507/MW2506	91/122
	MW0268/MW0269	617/164
Abi (type III/IV)	MW2234/MW2233	245/104
	MW0420/MW0421	89/131
HipBA (type II)	MW0980/MW0981	91/179
PTS (type II/IV)	MW1437/MW1436	133/107
	MW1004/MW1005	84/129
	MW0777/MW0776	78/67
	MW1150/MW1149	94/391
	MW1196/MW1195	83/34
	MW1292/MW1291	89/102
	MW1432/MW1431	71/87
	MW1164/MW1165	275/130
	MW0101	50
	MW0036	103
	MW1827	128

**Table 2:** The predicted seven PIs in the genome of *S. aureus*. Each PI has different number of TA systems and unique virulence genes.

PIs	Size (bp)	Number of TA Systems	Representative Toxins from TA Systems	Virulence Genes in PIs
Phi-Sa-2	45,550	11	MW1381, MW1394, MW1397, MW1403, MW1413, MW1419, MW1432, MW1434, MW1437, MW1440, MW1492	Panton–Valentine leukocidin
VSa-gamma	20,881	4	MW1042, MW1045, MW1056, MW1070	Alpha hemolysin, phenol soluble modulin
Phi-Sa-3	43,598	3	MW1888, MW1919, MW1928	Staphylokinase
VSa-beta	31,977	3	MW1725, MW1733, MW1779	Proteases
VSa-alpha	34,481	3	MW0302, MW0324, MW0405	--
VSa-3	14,380	2	MW0749, MW0771	Enterotoxins, exotoxin
VSa-4	2710	0		--

## Supplementary Material

The following is available online at http://www.mdpi.com/2072-6651/10/11/473/s1, Table S1: List of all bacterial strains, vectors, and primers used in this study. The lower case letters represent restriction enzyme sites.
