# Supplementary material for: Bioinformatics and Functional Assessment of Toxin-Antitoxin Systems in Staphylococcus aureus
Source: Toxins (Basel). 2018 Nov 14;10(11):473. doi: 10.3390/toxins10110473 (PMC6266405; doi:10.3390/toxins10110473)
Supplement: Supplementary file 1 [file toxins-10-00473-s001.pdf]

Supplementary Materials: Bioinformatics and Functional Assessment of Toxin-Antitoxin Systems in *Staphylococcus aureus*

Gul Habib, Qing Zhu and Baolin Sun

Table S1. List of all bacterial strains, vectors, and primers used in this study. The lower case letters represent restriction enzyme sites.

Strain	Relevant Genotype	Source
S. aureus strains		
S. aureus WT	Methicillin-resistant *S. aureus* MW2	NARSA
RN4220	*S. aureus* restriction modification deficient strain	NARSA
E. coli strains		
Trans T1	Clone host strain for transformation	TransGen
BL21(DE3)	Expression strain for recombinant proteins	TransGen
**Plasmids**		
pRMC2	Shuttle vector, anhydrotetracycline inducible, Amp ^{r}, Chl ^{r}	Addgene
pET28a (+)	Expression vector with a hexahistidine tag, Kan ^{r}	Addgene
**Primer name**	**Oligonucleotide (5′-3′)**	**Application**
Pet2329-F-NdeI	GCGcatatgGCTAGGTTAAATATTA	Toxin induction
Pet2329-R- EcoRI	GCGgaattcTTAATAATGGTATTTAC	Toxin induction
Pet2330-F-NdeI	GCGcatatgATTATTAAAAATTATTC	Antitoxin induction
Pet2330-R- XhoI	GCGctcgagTTATACATCTATATCTT	Antitoxin induction
Pet2380-F-NdeI	GCGcatatgAGCAATTACACGGTTAA	Toxin induction
Pet2380-R- EcoRI	GCGgaattcTTAATCATAATGTGACC	Toxin induction
Pet2381-F-NdeI	GCGcatatgATTATCACTAGCCCTA	Antitoxin induction
Pet2381-R- XhoI	GCGctcgagTTAAAGATTATCCCAATC	Antitoxin induction
Pet1992-F-NdeI	GCGcatatgATTAGACGAGGAGATGT	Toxin induction
Pet1992-R- EcoRI	GCGgaattcCTAATTTTTCTGGTGAG	Toxin induction
Pet1993-F-BamHI	GCGggatccATGTTATCTTTTAGTCA	Antitoxin induction
Pet1993-R- EcoRI	GCGgaattcTCATTCATTCGTTGAA	Antitoxin induction
Pet1419-F-NdeI	GCGcatatgAATAATCGTGAACAAA	Toxin induction
Pet1419-R- EcoRI	GCGgaattcCTAATACTCATTTTCCTG	Toxin induction
Pet1418-F-NdeI	GCGcatatgAGTATTAGTGTAGGAGA	Antitoxin induction
Pet1418-R- EcoRI	GCGgaattcTCACTCGTCCCCCTTAA	Antitoxin induction
PR1888-F-KpnI	GCGggtaccAGCCGTCTATTTGATATT	Toxin induction
PR1888-R-EcoRI	GCGgaattcTTAATCTAAAATAGCC	Toxin induction
PR2354-F-KpnI	GCGggtaccAGGAGGTGTATGACG	Toxin induction
PR2354-R-EcoRI	GCGgaattcTTATTTGTCACCTTT	Toxin induction
PR1381-F-KpnI	GCGggtaccAGGAGCAAACAAATG	Toxin induction
PR1381-R-EcoRI	GCGgaattcCTACCCTAAATCATTTG	Toxin induction
Biotn-F-67	ATCTCGAAAAATATTTTT	Gel shift assay
Biotn-R-67	TTCAAATTTACCTCCGTTTT	Gel shift assay
RT-F-*clpP*	CTAGGAGACATCAGTGAA	qRT-PCR
RT-R-*clpP*	CACTCATAGCGATAACAC	qRT-PCR
RT-F-*clpX*	CGTCGAAGAAGAATTAGC	qRT-PCR

RT-R-*clpX*	TGGTCCTAATTGTTGAATAC	qRT-PCR
Trans-F-36	GCGGAAATAGGTAAATT	Co-transcription
Trans-R-37	TATATTGTTCTATTGC	Co-transcription
Trans-F-29	ACAAAAGTAAATGATGA	Co-transcription
Trans-R-30	TCGTGATTGATACGTC	Co-transcription
